# Valproic acid induces differentiation and inhibition of proliferation in neural progenitor cells via the beta-catenin-Ras-ERK-p21^Cip/WAF1 ^pathway

**DOI:** 10.1186/1471-2121-9-66

**Published:** 2008-12-09

**Authors:** Gyung-Ah Jung, Ju-Yong Yoon, Byoung-San Moon, Dong-Hwa Yang, Hyun-Yi Kim, Sang-Hun Lee, Vitezslav Bryja, Ernest Arenas, Kang-Yell Choi

**Affiliations:** 1 National Research Laboratory of Molecular Complex Control, Department of Biotechnology, College of Life Science and Biotechnology, Yonsei University, Seoul, 120-752, Korea; 2 Department of Biochemistry, College of Medicine, Hanyang University, Seoul, 133-791, Korea; 3 Laboratory of Molecular Neurobiology, Department of Medical Biochemistry and Biophysics, Karolinska Institutet, S1-171 77 Stockholm, Sweden

## Abstract

**Background:**

Valproic acid (VPA), a commonly used mood stabilizer that promotes neuronal differentiation, regulates multiple signaling pathways involving extracellular signal-regulated kinase (ERK) and glycogen synthase kinase3β (GSK3β). However, the mechanism by which VPA promotes differentiation is not understood.

**Results:**

We report here that 1 mM VPA simultaneously induces differentiation and reduces proliferation of basic fibroblast growth factor (bFGF)-treated embryonic day 14 (E14) rat cerebral cortex neural progenitor cells (NPCs). The effects of VPA on the regulation of differentiation and inhibition of proliferation occur via the ERK-p21^Cip/WAF1 ^pathway. These effects, however, are not mediated by the pathway involving the epidermal growth factor receptor (EGFR) but via the pathway which stabilizes Ras through β-catenin signaling. Stimulation of differentiation and inhibition of proliferation in NPCs by VPA occur independently and the β-catenin-Ras-ERK-p21^Cip/WAF1 ^pathway is involved in both processes. The independent regulation of differentiation and proliferation in NPCs by VPA was also demonstrated *in vivo *in the cerebral cortex of developing rat embryos.

**Conclusion:**

We propose that this mechanism of VPA action may contribute to an explanation of its anti-tumor and neuroprotective effects, as well as elucidate its role in the independent regulation of differentiation and inhibition of proliferation in the cerebral cortex of developing rat embryos.

## Background

Valproic acid (VPA; 2-propyl-pentanoic acid) has been used for mood stabilization and the treatment of epilepsy for several decades [1]. VPA also exhibits potent *in vitro *and *in vivo *anti-tumor effects in leukemic cells, neuroblastomas, and gliomas [2-7]. VPA is a histone deacetylase (HDAC) inhibitor and plays a role in modifying chromatin structure and gene expression [8,9]. VPA has also been found to affect various signaling systems, including the extracellular signal-regulated kinase (ERK), protein kinase C (PKC), and the Wnt/β-catenin pathways [3,10,11]. VPA alters the Wnt/β-catenin signaling by directly or indirectly [12,13] inhibiting the activity of glycogen synthase kinase 3β (GSK3β).

VPA has been reported to regulate the differentiation and proliferation of various cells, including mesenchymal and hematopoietic stem cells, neuroblastoma cells, primary neurons, and neural progenitor cells (NPCs) [8,14-17]. VPA can also reduce the proliferation of neuroblastoma cells by induction of the cell cycle regulator p21^Cip/WAF1 ^[5,6], which is also known to be involved in the VPA-induced differentiation of hematopoietic cells [18]. However, the mechanism by which VPA regulates differentiation and proliferation is not understood.

We report here that 1 mM VPA induces differentiation and inhibits proliferation of NPCs by overcoming the effect of basic fibroblast growth factor (bFGF), a factor which inhibits the differentiation of NPCs [19,20]. VPA-induced activation of the ERK- p21^Cip/WAF1 ^pathway did not occur via the common pathway involving epidermal growth factor receptor (EGFR), an upstream component of the ERK pathway, as indicated by significant reduction in the level of EGFR in the presence of VPA. The level of Ras protein, another upstream component of the ERK pathway, was significantly increased by VPA treatment. This observation led us to conclude that VPA-induced ERK pathway activation occurs via an increase in the stability of Ras, mediated by Wnt/β-catenin signaling [21,22]. We also found that the common Ras-ERK-p21^Cip//WAF1 ^pathway is involved in generating the mutually exclusive phenotypes of differentiation and proliferation in NPCs and in brain tissue of the cerebral cortex of developing embryos.

## Results

### VPA overcomes the effects of bFGF on differentiation and proliferation in multipotent NPCs

**Figure 1:**
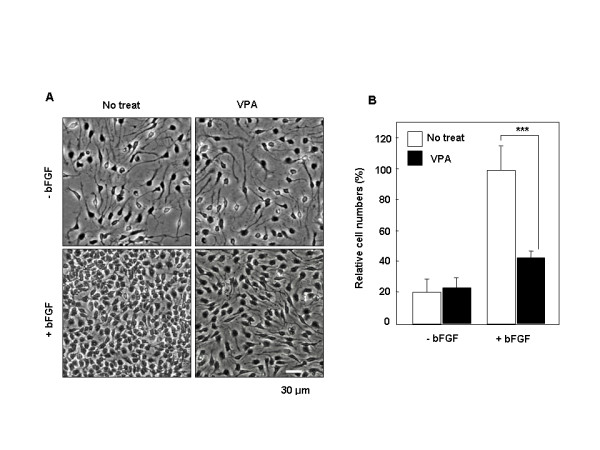
**Effect of VPA on morphology and proliferation of NPCs**. NPCs were treated with 1 mM VPA for 48 h in the presence or absence of 10 ng/ml bFGF. (A) Micrographs were taken at a magnification of 200×. (B) Cells were harvested and then counted using a hemocytometer. In three independent experiments, cells within the 230-μm square were counted and expressed as percent of the total number of cells in bFGF-treated cultures. Data represent mean ± SD of three separate experiments. P*** < 0.001 by one-way ANOVA followed by Newman-Keuls test.

Basic fibroblast growth factor (bFGF) is necessary for the maintenance of multipotency in neural progenitor cells [20] and is involved in the regulation of differentiation and growth in neuronal cells [23-25]. In agreement with earlier reports, we found that NPCs isolated from the cerebral cortex of E14 rat embryos underwent morphologic differentiation when grown in N2 medium alone (Figure 1A, upper left panel). The NPCs retain the capacity for self-renewal, as shown by their ability to form neurospheres, by a process of dissociation and reformation, for several passages in culture (see Additional file 1). The NPCs also retain the property of multipotency, as shown by their capacity to differentiate into primary neurons, oligodendrocytes, and astrocytes (see Additional file 1). The NPCs maintained an undifferentiated morphology when grown in the presence of 10 ng/ml bFGF (Figure 1A, lower left panel). The differentiation-suppressing and proliferative effects of bFGF were partly overcome by treatment with 1 mM VPA (Figure 1A, lower right panel). Cells treated with VPA and bFGF also exhibited more pronounced neurite outgrowth compared to cells treated with bFGF alone. The effect of VPA on morphologic differentiation in the presence of bFGF was dose-dependent (see Additional file 2). No evidence of cell toxicity was detected in the presence of 1 mM VPA and this concentration was used in the remaining experiments. Not only do NPCs grown in the presence of bFGF for 48 h remain morphologically undifferentiated, but the cells also had a high proliferation rate, as indicated by a five-fold increase in total cell number compared to untreated cultures (Figure 1B). In contrast, co-treatment with 1 mM VPA for 48 h reduced cell number by 60% compared to cells treated with bFGF alone (Figure 1B). VPA did not alter cell number in cultures grown in the absence of bFGF. Therefore, VPA induced both differentiation and inhibition of proliferation in NPCs by overcoming the anti-differentiation and pro-growth effects of bFGF.

### VPA induces both p21^Cip/WAF1 ^and Tuj1 via the Raf-MEK-ERK cascade in NPCs grown in the presence of bFGF

**Figure 2:**
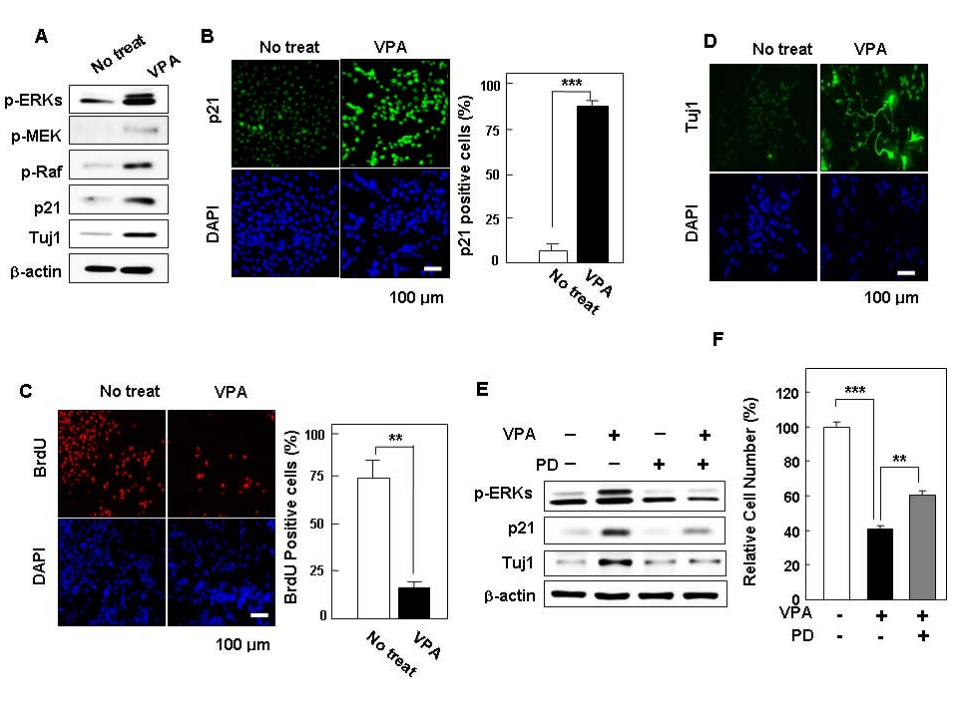
**Effect of VPA on activities of ERK pathway components and on expression of p21^Cip/WAF1 ^and Tuj1**. NPCs grown in 10 ng/ml bFGF were treated with 1 mM VPA for 48 h. (A) Whole-cell lysates were subjected to immunoblotting for analysis of the presence of p-ERK, p-MEK, p-Raf-1, p21^Cip/WAF1^, Tuj1, or β-actin. Alternatively, NPCs were processed for immunofluorescent labeling of (B) p21^Cip/WAF1 ^(green), (C) BrdU (red), or (D) Tuj1 (green). Nuclei were counterstained with DAPI (blue). All images are 200× magnifications. The percentage of cells that exhibited nuclear p21^Cip/WAF1 ^in the presence and absence of VPA is presented, along with the percentage of BrdU-positive and Tuj1-positive cells. Error bars indicate the standard deviations of three independent experiments. Data represent mean ± SD of three separate experiments. P*** < 0.001, P** < 0.01 by Student's *t*-test. (B-C). Effects of inhibition of ERK pathway on p21^Cip/WAF1 ^and Tuj1 induction by VPA. (E) NPCs grown in the presence of 10 ng/ml bFGF were treated with different combinations of 20 μM PD98059 and 1 mM VPA. (F) NPCs grown in the presence of 10 ng/ml bFGF were treated as in (E). In three independent experiments, cells within the 230-μm square were counted and expressed as a percentage of the total number of cells. Values represent the mean ± SD of three separate experiments. P** < 0.01, P*** < 0.001 by one-way ANOVA followed by Newman-Keuls test.

The ERK pathway is involved in both positive and negative regulations of cell growth depending upon the intensity and duration of the signals [26-28] and VPA can activate the ERK pathway in primary neurons in culture [29,30]. To investigate the potential involvement of the ERK pathway in VPA-induced differentiation and growth inhibition in bFGF-treated NPCs, the activation status of Raf-1, MEK, and ERK was examined. Immunoblot analysis revealed that VPA treatment elicited an increase in ERK activity, as indicated by an increase in the levels of phosphorylated ERK (p-ERK), in NPCs grown in bFGF. The levels of phosphorylated MEK (p-MEK) and Raf-1 (p-Raf-1) were also increased in NPCs in which differentiation had been induced and proliferation had been inhibited by treatment with VPA (Figure 2A). Induction of the cell cycle regulator p21^Cip/WAF1 ^is an important indicator of ERK pathway-induced inhibition of proliferation [28,31]. In accordance with its anti-proliferative effect, VPA elicited an increase in p21^Cip/WAF1 ^levels in NPCs grown in the presence of bFGF (Figure 2A). Immunocytochemical analysis of NPCs revealed that the induction of p21^Cip/WAF1 ^by VPA was accompanied by accumulation of p21^Cip/WAF1 ^in the cell nuclei (Figure 2B; left panel). The number of cells containing positive nuclear staining of p21^Cip/WAF1 ^increased from 5% to 87.5% in response to VPA treatment (Figure 2B, right panel). In contrast, the percentage of proliferating cells, which incorporated BrdU into their nuclei, was reduced from 73% to 15% in response to treatment with 1 mM VPA (Figure 2C). Immunoblot analysis revealed that the level of Tuj1, a specific marker of immature neurons, increased in NPCs grown in the presence of VPA and bFGF (Figure 2A). Immunocytochemical analysis confirmed these findings and also showed marked, enhanced neurite outgrowth in cells showing significantly increased levels of Tuj1 (Figure 2D).

To ascertain whether the ERK pathway mediates p21^Cip/WAF1 ^induction and neuronal differentiation in response to VPA, we measured the effect of inhibition of the Ras-ERK pathway on the induction of p21^Cip/WAF1 ^and Tuj1 by VPA. Induction of p21^Cip/WAF1 ^by VPA was significantly reduced when ERK activation was inhibited by the MEK inhibitor PD98059 (Figure 2E). Induction of Tuj1 by VPA was also reduced by PD98059. The VPA-induced activation of the ERK pathway in the inhibition of growth of NPCs in the presence of bFGF was confirmed by the observation of re-increase of cell numbers by treatment with PD98059 in the presence of VPA (Figure 2F). We did not observe any growth-stimulatory effect of PD98059 when NPCs were grown in the absence of bFGF; rather, cell number was somewhat reduced by PD98059 treatment (see Additional file 3). In summary, VPA induces differentiation and inhibition of proliferation via the Ras→MEK→ERK pathway in NPCs grown in the presence of bFGF.

### p21^Cip/WAF1 ^mediates VPA-induced differentiation and inhibition of proliferation in NPCs grown in the presence of bFGF

**Figure 3:**
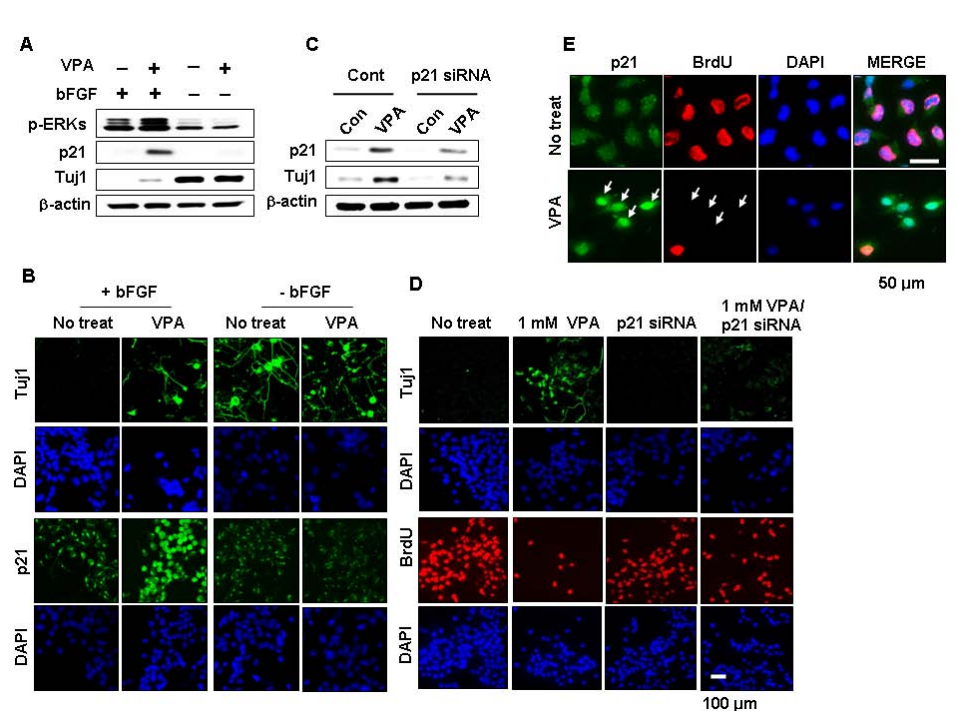
**Effects of bFGF and p21^Cip/WAF1 ^siRNA on regulation of the VPA-induced differentiation and inhibition of proliferation in NPCs (A-B)**. NPCs were treated with 1 mM VPA for 48 h in the presence or absence of 10 ng/ml bFGF. (C-D) NPCs were transfected with 100 nM p21^Cip/WAF1 ^siRNA prior to treatment with 1 mM VPA for 48 h in the presence of 10 ng/ml bFGF. Whole-cell lysates were subjected to immunoblotting to detect p21^Cip/WAF1^, Tuj1, or β-actin. Alternatively, cells were processed for immunofluorescent labeling to detect the presence of Tuj1 (green), p21^Cip/WAF1 ^(green), or BrdU (red). Nuclei were counterstained with DAPI. Images are 400× magnifications. (E) NPCs were treated with 1 mM VPA for 48 h in the presence of 10 ng/ml bFGF. Cells were processed for immunofluorescent labeling to detect the presence of p21^Cip/WAF1 ^(green) or BrdU (red). Nuclei were counterstained with DAPI. Magnification is 1200×.

VPA-induced ERK activation and p21^Cip/WAF1 ^induction were not observed in the Tuj1-induced, differentiated NPCs grown in the absence of bFGF, as shown by both biochemical (Figure 3A) and immunocytochemical (Figure 3B) analyses. In these cultures, the number of BrdU-positive cells was relatively low and was not changed by VPA treatment (see Additional file 4). To determine the role of p21^Cip/WAF1 ^in VPA-induced differentiation and inhibition of proliferation, we analyzed the effect of p21^Cip/WAF1 ^siRNA. Stimulation of neuronal differentiation and inhibition of proliferation in NPCs by VPA treatment were diminished by p21^Cip/WAF1 ^siRNA as shown by both biochemical (Figure 3C) and immunocytochemical (Figure 3D) analyses. Inhibition of proliferation, one of the effects of VPA on NPCs as assessed by BrdU incorporation, was also released after treatment with p21^Cip/WAF1 ^siRNA (Figure 3D; quantitative data are shown in Additional file 5). The majority of cells exhibiting nuclear accumulation of p21^Cip/WAF1 ^after VPA treatment were also BrdU-negative, underscoring the important anti-proliferative role of p21^Cip/WAF1 ^(Figure 3E).

### VPA activates the ERK-p21^Cip/WAF1 ^pathway and induces differentiation and inhibition of proliferation via β-catenin-mediated accumulation of Ras in NPCs grown in the presence of bFGF

**Figure 4:**
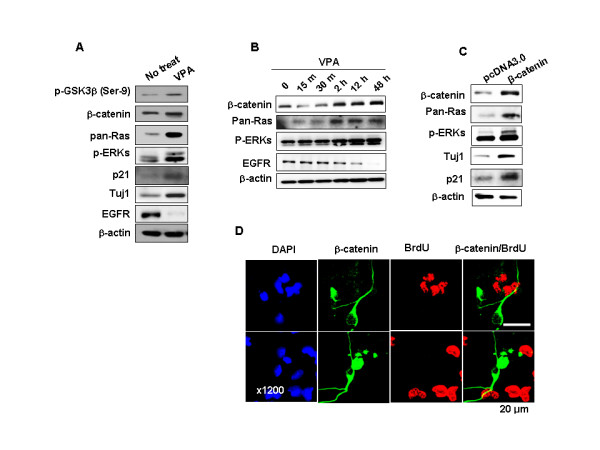
**Effects of VPA or β-catenin on EGFR, β-catenin, and Ras regulation**. (A-B) NPCs grown in 10 ng/ml bFGF were treated with 1 mM VPA for 48 h or different periods of time. (C-D) NPCs were transfected with pcDNA3.0 or Flag-β-catenin-pcDNA3.0 and grown in the presence of 10 ng/ml bFGF. (A-C) Whole-cell lysates were then subjected to immunoblotting for detection of p-GSK3β (Ser-9), β-catenin, Pan-Ras, p-ERK, p21^Cip/WAF1^, Tuj1, EGFR, or β-actin. (D) Immunofluorescent labeling was performed for Flag or BrdU. Nuclei were counterstained with DAPI.

The epidermal growth factor receptor (EGFR) most often mediates activation of the ERK pathway by extracellular stimuli [32]. Although, in this study, the ERK pathway was activated by VPA, we were surprised to find that EGFR levels were reduced by VPA treatment in NPCs grown in the presence of bFGF (Figure 4A). The VPA-induced decrease in EGFR levels was convincingly seen in the time course of VPA treatment after treatment with 1 mM VPA (Figure 4B). VPA regulates the Wnt/β-catenin pathway by direct binding and inhibition of GSK3β [12,13]. In this study, we also observed an increase in the level of phospho-Ser-9-GSK3 (p-GSK3β), an inactive form of GSK3β [14], and β-catenin following VPA treatment (Figure 4A). It is also known that Ras protein levels are subject to regulation by the Wnt/β-catenin pathway in several cell types, including primary hepatocytes, and β-catenin has been identified as a mediator in that process [21,22]. Here, we found that the level of Ras protein was significantly up-regulated in NPCs treated with VPA, leading to an increase in p-GSK3β and its target, β-catenin (Figure 4A). The role of β-catenin in the regulation of Ras was further indicated by similar patterns of increase in Ras and β-catenin levels during the time course of VPA treatment (Figure 4B).

To identify the role of β-catenin in the regulation of the Ras-ERK-p21^Cip/WAF1 ^pathway and subsequent stimulation of differentiation and inhibition of proliferation induced by VPA in NPCs, we measured the effect of overexpression of β-catenin. Overexpression of β-catenin increased ERK activity and Ras protein level and induced expression of p21^Cip/WAF1 ^and Tuj1 (Figure 4C). Cells overexpressing β-catenin were morphologically differentiated and BrdU-negative (Figure 4D). Therefore, β-catenin is an important factor for both differentiation and inhibition of proliferation in cortical E14 NPCs and those physiological responses are simultaneously and independently controlled by β-catenin.

**Figure 5:**
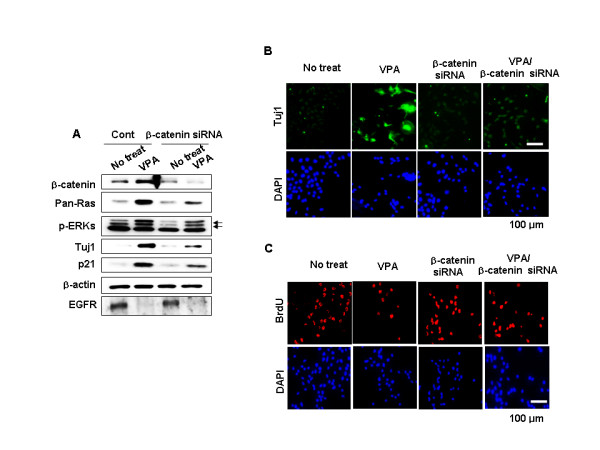
**Effects of β-catenin knockdown on Ras-ERK pathway activation and VPA-induced differentiation and inhibition of proliferation**. NPCs were transfected with 100 nM control siRNA or β-catenin siRNA prior to treatment with 1 mM VPA for 48 h. (A) Whole-cell lysates were subjected to immunoblotting to detect presence of β-catenin, Pan-Ras, p-ERK, p21^Cip/WAF1^, Tuj1, EGFR, or β-actin. (B-C) Immunofluorescent labeling of Tuj1or BrdU. Nuclei were counterstained with DAPI.

The role of β-catenin in the VPA-induced expression of Tuj1 and p21^Cip/WAF1 ^in NPCs was further investigated by measurement of the effects of β-catenin siRNA. We observed that the VPA-induced increase in Ras was reduced following β-catenin knockdown (Figure 5A). ERK activation, and subsequent induction of Tuj1 and p21^Cip/WAF1^, was also reduced by β-catenin knockdown (Figure 5A), indicating that β-catenin, at least in part, mediates the effect of VPA on the activation of the ERK-p21^Cip/WAF1^-Tuj1 pathway via stabilization of Ras. VPA-induced reduction in EGFR levels was not affected by β-catenin knockdown. The role of β-catenin in VPA-induced differentiation and inhibition of proliferation was also examined (Figure 5B and 5C). Immunocytochemical analysis revealed that β-catenin siRNA significantly reduced VPA-mediated induction of Tuj1 in NPCs (Figure 5B). In contrast, the number of BrdU-positive cells, which initially decreased in the presence of VPA, was marginally increased by VPA treatment following silencing of β-catenin (Figure 5C; quantitative data are shown in Additional file 6).

### VPA independently induces differentiation and inhibition of proliferation in NPCs in the developing rat brain

**Figure 6:**
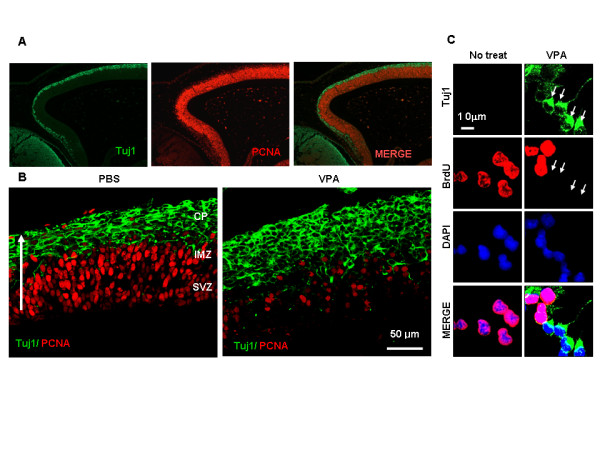
**Effects of VPA on differentiation and proliferation in cerebral cortex of the developing embryo**. (A) Coronal sections of E15.5 rat embryo cerebral cortex immunofluorescently labelled for Tuj1 (green) and PCNA (red). (B) Rats at E13.5 of gestation were intravenously injected with 200 mg/kg VPA or PBS at 0 and 24 h and sacrificed. Immunofluorescence labeling of Tuj1 (green) and PCNA (red) was performed on coronal sections of embryonic brains of the E15.5 rat. The three layers of the cerebral cortex – the cortical plate (CP), the intermediate zone (IMZ), and the subventricular zone (SVZ) above the lateral ventricle (LV)- can be distinguished. The white arrow indicates the direction of migration of the differentiating cells (bar = 25 μm). (C) NPCs from E14 embryos were incubated in the presence of 10 ng/ml bFGF in the presence or absence of 1 mM VPA for 48 h. Immunofluorescence labeling was performed with anti-Tuj1 and anti-BrdU. Nuclei were counterstained with DAPI.

To understand the physiological relevance of the effects of VPA observed *in vitro*, we administered VPA to E13.5 rat embryos and examined the effect on differentiation and proliferation in the cerebral cortex. In the absence of VPA, the immunofluorescent labeling patterns of proliferating cell nuclear antigen (PCNA) and Tuj1 in the cerebral cortex of the developing rat embryo were distinct and non-overlapping (Figure 6A and 6B). In control embryos injected with PBS alone, Tuj1 staining was predominantly localized to the cortical plate (CP) area, where differentiated neuronal cells reside. However, PCNA staining was primarily localized to the subventricular zone (SVZ) and the intermediate zone (IMZ), where neural stem/progenitor cells reside (Figure 6B; left panel). The number of cells that had differentiated into neurons was significantly increased by VPA, to the extent that both the area occupied by Tuj1-positive cells and the intensity of the Tuj1 staining were significantly increased in the CP region (Figure 6B). In contrast, the intensity and number of PCNA-positive cells were significantly reduced by VPA in the SVZ and IMZ of the cerebral cortex (Figure 6B); of the cerebral cortical cells induced to differentiate by VPA treatment, none were PCNA-positive. Demonstration of differentiation and proliferation in cells of the VPA-treated cerebral cortex was also carried out *in vitro*, and similar distinct patterns of Tuj1 and BrdU staining were observed in the VPA-treated NPCs grown in the presence of bFGF (Figure 6C).

## Discussion

In the present study, we found that NPCs undergo neuronal differentiation and inhibition of proliferation following treatment with 1 mM of the commonly prescribed antiepileptic drug VPA [1]. We chose 1 mM VPA because this concentration is non-toxic to the NPCs in our current study and to hippocampal neuronal progenitors [2]. The VPA amount applied to the animals, 200 mg/kg, is identical to that used to achieve whole-brain levels of 1.0–1.5 mM by chronic application [1].

We investigated the mechanism by which VPA regulates differentiation and inhibition of proliferation. The role of VPA in the exclusive *in vivo *regulation of differentiation and proliferation of NPCs suggest an application for VPA in the production of functional neurons for therapeutic use in patients. Our study also provides a mechanism that may aid in validating the proposed use of VPA as an anti-tumor and neuroprotective agent [3,4,33]. VPA induced both differentiation and inhibition of proliferation in NPCs by overcoming the effect of bFGF, a factor which promotes growth/proliferation and suppresses differentiation, through the common ERK-p21^Cip/WAF1 ^pathway. The VPA-induced inhibition of proliferation was suppressed by the MEK inhibitor PD98059, indicating a role for activation of the ERK pathway in the inhibition of proliferation by VPA. Participation of the ERK pathway in inhibition of proliferation is frequently accompanied by induction of the cell cycle inhibitory factor p21^Cip/WAF1 ^[6,31,34,35]; p21^Cip/WAF1 ^was also induced in the VPA-treated NPCs. The role of p21^Cip/WAF1 ^in inhibition of proliferation by VPA is also shown by release of the VPA-induced inhibition of proliferation by p21^Cip/WAF1 ^siRNA. The role of p21^Cip/WAF1 ^as a potent anti-proliferation factor was further shown by loss of BrdU incorporation in most cells in which p21^Cip/WAF1 ^had been induced by VPA.

A significant decrease in the level of Tuj1 following siRNA-mediated p21^Cip/WAF1 ^knockdown, demonstrated by both biochemical and immunocytochemical analyses, suggests that p21^Cip/WAF1 ^may also be involved in the regulation of differentiation and inhibition of proliferation. It is clear that both inhibition of proliferation and differentiation of NPCs stimulated by VPA occur through the ERK pathway-dependent induction of p21^Cip/WAF1^.

We have shown that activation of the ERK-p21^Cip/WAF1 ^pathway by VPA did not occur via the segment of the pathway involving EGFR but via the segment involving β-catenin. Although EGFR has been identified as a target of Wnt/β-catenin in liver [36], EGFR was reduced by VPA in NPCs. The mechanism by which *EGFR *transcription is inhibited by VPA is unknown; however, it has been established that *EGFR *transcription is repressed by bone morphogenetic protein 4 (BMP4), an alternative transcription target of β-catenin [37], in NPCs [38,39]. We observed significant induction of BMP4 in the VPA-treated NPCs grown in the presence of bFGF (data not shown). These data suggest that the VPA-induced decrease in EGFR in NPCs may be acquired through induction of BMP4.

VPA directly inhibits GSK3β resulting in activation of the β-catenin signaling pathway [33,40,41] and β-catenin is, in turn, involved in regulation of the ERK pathway [21,22]. Evidence for the role of β-catenin in VPA-induced activation of the ERK-p21^Cip/WAF1 ^pathway, and subsequent effects on differentiation and inhibition of proliferation in NPCs, was seen in the reduction of the effects of VPA, including ERK activation and induction of p21^Cip/WAF1 ^and Tuj1, following siRNA-mediated β-catenin knockdown. The β-catenin-mediated activation of the ERK-p21^Cip/WAF1 ^pathway following VPA treatment may be attributed to upregulation of Ras, suggested by the increase in the level of Ras seen after β-catenin overexpression. The VPA-induced increase in the level of Ras may be due to the stabilization of β-catenin as a result of inhibition of GSK3?. Increases in Ras following modulation of the Wnt/β-catenin signaling pathway have been demonstrated in various cell types, including primary hepatocytes, and β-catenin has been identified as an important mediator of that process [21,22]. Regulation of Ras protein levels by the Wnt/β-catenin system is mediated by polyubiquitination and proteasomal degradation [Kim et al., 2008, Journal of Cell Science, In print]. Differentiation and proliferation occur independently in the cerebellar cortex of the developing embryo [42-44]; however, the mechanism(s) underlying the differential regulation of the two processes has not been described. In this study, we found that differentiation and proliferation occurred independently in regions of the developing brain of embryos treated with VPA. We saw no BrdU-positive cells among Tuj1-positive NPCs following VPA treatment. We also did not observe any proliferating cells among differentiated NPCs stimulated to differentiate by β-catenin overexpression or by VPA treatment to express increased levels of p21^Cip/WAF1^. These results indicate that mutually exclusive patterns of differentiation and proliferation during neuronal differentiation and development may be regulated via the common Ras-ERK-p21^Cip/WAF1 ^pathway involving β-catenin.

However, the effects of VPA, particularly its effect on proliferation, were modest or only partially inhibited by increases in p21^Cip/WAF1 ^or siRNA-mediated β-catenin knockdown in several different cases. These results indicate the possibility that VPA-induced differentiation and inhibition of proliferation occur in part via different routes, including, e.g., the pathway affected by inhibition of HDAC [8]. Although we improved the efficiency of transfection by making modifications to the standard method, the limited effectiveness of siRNAs in general may also be a contributing factor in the weak effects seen on differentiation and inhibition of proliferation (see Additional file 7). The concomitant stimulation of differentiation and inhibition of proliferation in NPCs and the developing rat embryo by VPA treatment indicate potential utility for VPA in the treatment of neuroblastomas [45] and/or in neuronal regeneration.

## Conclusion

Our results suggest that VPA induces neuronal differentiation and inhibition of proliferation of cortical NPCs at least partly via the Ras-ERK-p21^Cip/WAF1^-pathway mediated by β-catenin. We propose that this mechanism of VPA action may contribute to an explanation of its anti-tumor and neuroprotective effects, as well as elucidate its role in the independent regulation of differentiation and proliferation in the cerebral cortex of developing rat embryos. Regulation of Ras stability in neuronal differentiation and inhibition of proliferation may indicate a new role of the protooncoprotein Ras in the differentiation of stem cells and in development.

## Methods

### Primary cerebral cortical progenitor cell culture, transfection, and VPA treatment

Sprague-Dawley (SD) rats were purchased from KOATECH (Gyeonggi Do, Korea). All animal procedures were approved by the Institutional Review Board of Severance Hospital, Yonsei University College of Medicine. NPCs were isolated from the cerebral cortex of E14 SD rats as described previously [26]. The isolated cortical cells were plated in dishes coated with 15 μg/ml poly-L-ornithine and 10 μg/ml fibronectin (Sigma-Aldrich, St. Louis, MO) and cultured in N2 medium [DMEM:F12 (1:1) (Invitrogen) containing 100 μM putrescine, 30 nM selenite, 20 nM progesterone, 1.55 mg/ml D-(+)-glucose, 25 μg/ml insulin, 0.1 μg/ml apo-transferrin (Sigma-Aldrich), 0.5 mM Glutamax, 100 IU/ml penicillin, and 100 μg/ml streptomycin] containing 10 ng/ml bFGF (basic Fibroblast Growth Factor; Invitrogen, Carlsbad, CA) in a humidified atmosphere of 95% air/5% CO_2 _at 37°C. NPCs were plated at 4 – 5 × 10^5 ^cells per dish in a 60-mm culture dish and grown to 40% confluence, cells were treated with 0.5 – 10 mM (most often 1 mM) VPA for 48 h. To observe the effect of inhibition of MEK, some cultures were co-treated with 20 μM PD98059 (2'-amino-3'-methoxyflavone) (A G. Scientific Inc, San Diego, CA) in addition to VPA. To test the effects of overexpression or knockdown, vectors or siRNAs were transfected into NPCs using Lipofectamine TM 2000 (Invitrogen). Transfections were performed in N2 medium without antibiotics, followed by growth in N2 medium containing 50 IU/ml penicillin and 50 μg/ml streptomycin, prior to treatment with VPA. Cultures were photographed using the ECLIPS TE2000-U Fluorescent microscope (Nikon, Tokyo, Japan) equipped with a digital CCD camera (Diagnostic Instruments, Inc., Sterling Heights, MI).

### VPA Treatment of embryos

Beginning on embryonic day 13.5 (E13.5) of gestation, rats were intravenously injected with 200 mg/kg VPA (Acros Organics, Belgium) or phosphate-buffered saline (PBS) twice at 24 h intervals, and provided with water containing 15 g/l VPA for 48 h. On day15.5 embryos were collected for subsequent immunohistochemical analyses.

### Immunoblot Analysis

Cells were rinsed with ice-cold PBS and lysed in 1× Laemmli buffer (0.14 M Tris, pH 6.8, containing 2.4 M glycerol, 0.21 M sodium dodecyl sulfate, and 0.3 mM bromophenol blue). Cell lysates were heated at 100°C for 10 min and separated by electrophoresis through 8 – 12% SDS-polyacrylamide gels. Following electrophoretic transfer of proteins to nitrocellulose, membranes were blocked in 5% nonfat dry milk in PBS containing 0.1% (v/v) Tween 20 for 30 min at 25°C followed by incubation with anti-p-ERK, anti-p-MEK, anti-p-GSK3β (Ser-9) (Cell Signaling Biotechnology, Beverly, MA), anti-β-catenin (rabbit polyclonal antibody produced in this laboratory), anti-p21^Cip/WAF1^, anti-EGFR (Santa Cruz Biotechnology, Santa Cruz, CA), anti-p-Raf-1 (Ser-338), anti-pan-Ras (Upstate Biotechnology, Lake Placid, NY), anti-Tuj1 (Covance, Princeton, NJ), or anti-β-actin (Abcam Ltd, MA) antibody followed by horseradish peroxidase-conjugated secondary anti-rabbit, anti-rat (Calbiochem, La Jolla, CA), or anti-mouse IgG (Bio-Rad Laboratories, Hercules, CA). Protein bands were visualized using enhanced chemiluminescence (ECL; Amersham, Inc, UK) and the LAS-3000 imaging system (Fujifilm, Tokyo, Japan).

### Immunocytochemistry and Immunohistochemistry

For immunocytochemistry, NPCs were plated on coverslips coated with poly-L-ornithine and fibronectin in 24-well plates at a density of 2 – 2.5 × 10^4 ^cells/well. When cultures reached 30% confluence VPA was added to the medium. Where indicated, the cells had been transfected with 100 nM p21^Cip/WAF1 ^siRNA or β-catenin siRNA for 12 h before VPA treatment. For the proliferation assay, cultures were incubated with 25 μM BrdU (Sigma-Aldrich) for 2.5 h before fixation. Cells were fixed in 70% ethanol for 30 min, washed three times with PBS, and permeabilized by incubation in 0.1% Triton X-100 in PBS for 30 min. To measure BrdU incorporation, cells were incubated in 2 M HCl for 30 min and washed three times with PBS. The cells were incubated in blocking solution (10% normal goat serum in PBS) for 30 min followed by incubation with anti-Tuj1, anti-BrdU (Dako Co., Carpinteria, CA), anti-Flag (Sigma-Aldrich), or anti-p21^Cip/WAF1 ^in blocking solution at 4°C overnight. Cells were washed three times in PBS and incubated with Alexa Fluor 488- or Alexa Fluor 555-conjugated IgG secondary antibody (Molecular Probes, Eugene, OR) at room temperature for 1 h. Nuclei were counterstained by incubation in 1 μg/ml DAPI (4',6-diamidino-2-phenylindole; Boehringer Mannheim, Mannheim, Germany) for 10 min followed by exhaustive washing in distilled water. Coverslips were mounted in GelMount (Biomeda, Foster City, CA). Fluorescent labeling was observed using a Radiance 2100 multi-photon imaging system (Bio-Rad Laboratories) and LSM510META (Carl Zeiss, Germany) at excitation wavelengths of 488 nm (Alexa Fluor 488), 543 nm (Alexa Fluor 555), or 405 nm (DAPI). Approximately 300–400 cells were counted in each of three independent experiments to quantify relative p21^Cip/WAF1^-, Tuj1-, or BrdU- positive cells. For histological analyses, embryos were fixed in 10% neutral buffered formalin for 48 h, dehydrated by serial immersion in alcohols, cleared in xylene, and embedded in paraffin. Four-micron sections were cut using a RM2245 microtome (Leica Microsystems Wetzlar, Germany). Immunohistochemical analysis was performed using Alexa Fluor 555- and 488-conjugated IgG secondary antibodies. Antigen retrieval was performed using citrate buffer, pH 6.0. All incubations were carried out in humidified chambers in the dark. Immunofluorescent labeling of tissue sections was performed using the staining procedure described above.

### Vector and siRNAs

Flag-β-catenin-pcDNA3.0 was obtained from Dr. Eric R. Fearon of the University of Michigan School of Medicine. Small interfering RNAs (siRNAs) targeting rat p21^Cip/WAF1 ^(GenBank accession number NM_080782) and β-catenin (GenBank accession number NM_053357) mRNAs were designed using the template design tool (Ambion, Austin, TX). The *p21*^*Cip/WAF1 *^target sequences are 5'-AGACCAGCCUAACAGATTTTT-3' (431–459) and 5'-GAACGGTGGAACTTTGACTTT-3' (136–154). *β-catenin *target sequences are 5'-AAGGCTTTTCCCAGTCCTTCA-3' (203–223) and 5'-AAGATGATGGTGTGCCAAGTG-3' (1303–1323). β-catenin and p21^Cip/WAF1^siRNAs were synthesized by Samchully Pharm Co., LTD (Gyeonggi do, Korea).

## Abbreviations

bFGF: basic fibroblast growth factor; BMP4: bone morphogenetic protein 4; CP: cortical plate; ERK: extracellular signal-regulated kinase; GSK3β: glycogen synthase kinase3β; HDAC: histone deacetylase; IMZ: intermediate zone; NPC: neural progenitor cells; Tuj1: class III β-tubulin; PCNA: proliferating cell nuclear antigen; SVZ: subventricular zone; siRNA: small interfering RNA; VPA: valproic acid, 2-propyl-pentanoic acid.

## Authors' contributions

GAJ, JYY, BSM, DHY, and HYK carried out the experiments. KYC coordinated and wrote the manuscript. SHL, VB, and EA provided reagents and techniques and approved the manuscript.

## Supplementary Material

Additional file 1 **NPCs isolated from E14 embryos retain the capacity to form neurospheres and to differentiate into primary neurons, oligodendrocytes, and astrocytes**. NPCs were isolated as described in Methods. (A) Neurospheres were established as previously described [46] and maintained in N2 medium containing bFGF (10 ng/ml) and epidermal growth factor (EGF, 20 ng/ml). Neurospheres were dissociated and re-formed for two passages (P1 and P2, respectively). Neurospheres were subjected to immunocytochemical analyses using anti-Tuj1, anti-glial fibrillary acidic protein (GFAP), or anti-O4 (oligodendrocyte marker O4) to detect primary neurons, astrocytes, and oligodendrocytes, respectively. Click here for file

Additional file 2 **Effects of different concentrations of VPA on morphological differentiation and proliferation of NPCs. NSCs were isolated from rat cerebral cortex at embryonic day 14 (E14) and cultured in N2 medium supplemented with 10 ng/ml bFGF as described in Methods**. At 40% confluence, the cultures were treated with 0, 0.5, 1, 2, 3, or 10 mM VPA and incubated for an additional 48 h. Micrographs were taken using a Nikon ECLIPSE TE2000-U fluorescence microscope at 200× magnification. Click here for file

Additional file 3 **Effects of MEK inhibitor PD98059 on the morphological differentiation and proliferation of NPCs. NPCs were grown in medium containing 10 ng/ml bFGF**. Medium was then replaced with fresh medium containing 10 ng/ml bFGF, 20 μM PD98059, and/or 1 mM VPA, as indicated, and cultures were incubated for an additional 48 h. Cultures were photographed and relative numbers of cells were estimated as described in Figure 1. Click here for file

Additional file 4 **Effects of bFGF and VPA on proliferation of NPCs**. This experiment is similar to that shown in Figure 3B. Cells were processed for immunofluorescent labeling of BrdU (red). Nuclei were counterstained with DAPI (blue). Images are 100× magnifications. Right panel: Estimate of the percentage of BrdU-positive cells. Error bars indicate the standard deviation of three independent experiments. Click here for file

Additional file 5 **Effects of p21^Cip/WAF1 ^siRNA on VPA-induced inhibition of proliferation of NPCs grown in the presence of bFGF**. This figure shows the quantitative data for Figure 3D (lower panel). Relative numbers of BrdU-positive cells were quantified. Error bars indicate the standard deviation of three independent experiments. Click here for file

Additional file 6 **Effects of ß-catenin siRNA on VPA-induced proliferation of NPCs grown in the presence of bFGF**. This  figure  shows  the  quantitative  data  for  Figure  5C.    Relative numbers of BrdU-positive cells were quantified.    Error bars indicate the standard deviation of three independent experiments. Click here for file

Additional file 7 **Measurements of NPC transfection efficiencies.** NPCs were  grown  in N2 medium  containing bFGF  (10 ng/ml),  and transfected  with  0,  0.5,  1,  2,  or  3  µg  pEGFP-C1  (Clontech)  as  described  in Methods.    Percentages  of  GFP-positive  cells  were  estimated  24  h  after transfection.    Error  bars  indicate  the  standard  deviation  of  three  independent experiments. Click here for file
