# Supplementary figures and images for: Valproic acid induces differentiation and inhibition of proliferation in neural progenitor cells via the beta-catenin-Ras-ERK-p21Cip/WAF1 pathway
Source: BMC Cell Biol. 2008 Dec 9;9:66. doi: 10.1186/1471-2121-9-66 (PMC2639384; doi:10.1186/1471-2121-9-66)

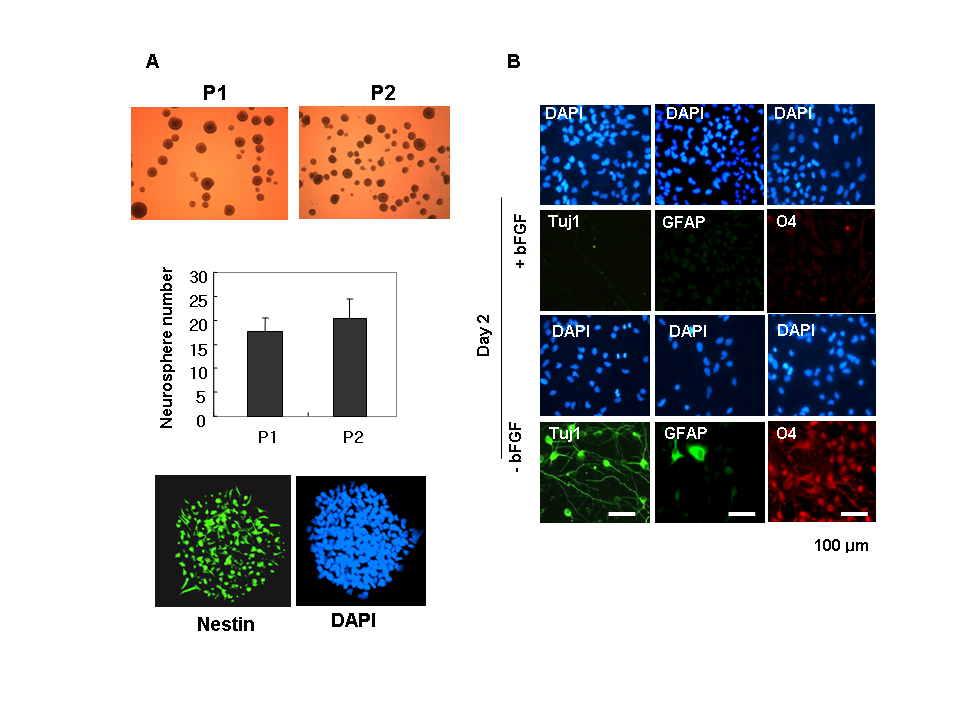

Supplement: Additional file 1 — NPCs isolated from E14 embryos retain the capacity to form neurospheres and to differentiate into primary neurons, oligodendrocytes, and astrocytes. NPCs were isolated as described in Methods. (A) Neurospheres were established as previously described [46] and maintained in N2 medium containing bFGF (10 ng/ml) and epidermal growth factor (EGF, 20 ng/ml). Neurospheres were dissociated and re-formed for two passages (P1 and P2, respectively). Neurospheres were subjected to immunocytochemical analyses using anti-Tuj1, anti-glial fibrillary acidic protein (GFAP), or anti-O4 (oligodendrocyte marker O4) to detect primary neurons, astrocytes, and oligodendrocytes, respectively. [file 1471-2121-9-66-S1.tiff]

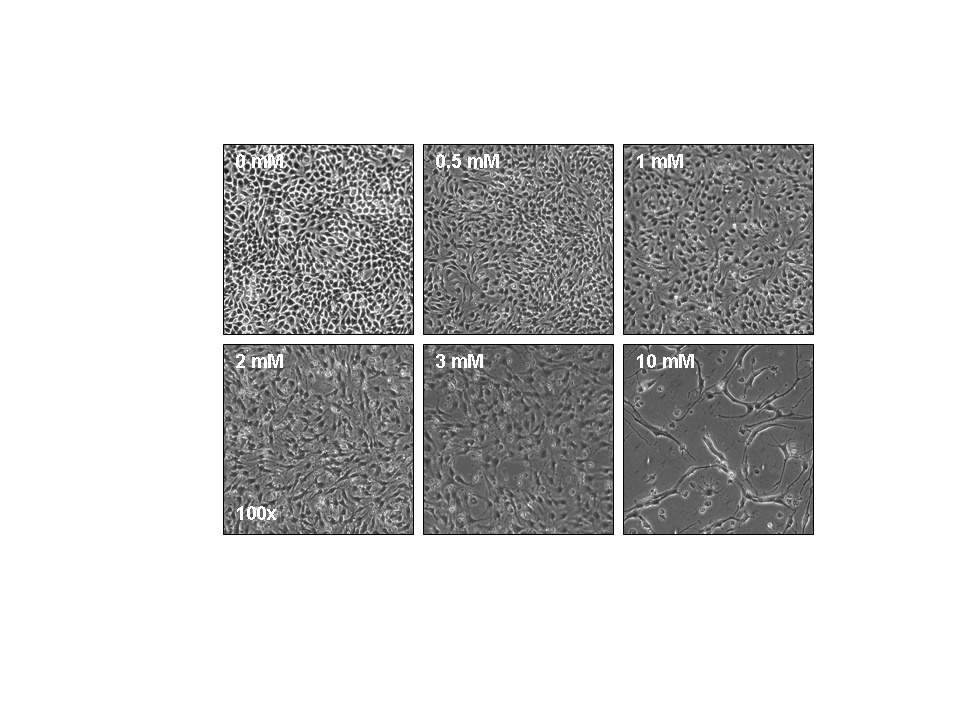

Supplement: Additional file 2 — Effects of different concentrations of VPA on morphological differentiation and proliferation of NPCs. NSCs were isolated from rat cerebral cortex at embryonic day 14 (E14) and cultured in N2 medium supplemented with 10 ng/ml bFGF as described in Methods. At 40% confluence, the cultures were treated with 0, 0.5, 1, 2, 3, or 10 mM VPA and incubated for an additional 48 h. Micrographs were taken using a Nikon ECLIPSE TE2000-U fluorescence microscope at 200× magnification. [file 1471-2121-9-66-S2.tiff]

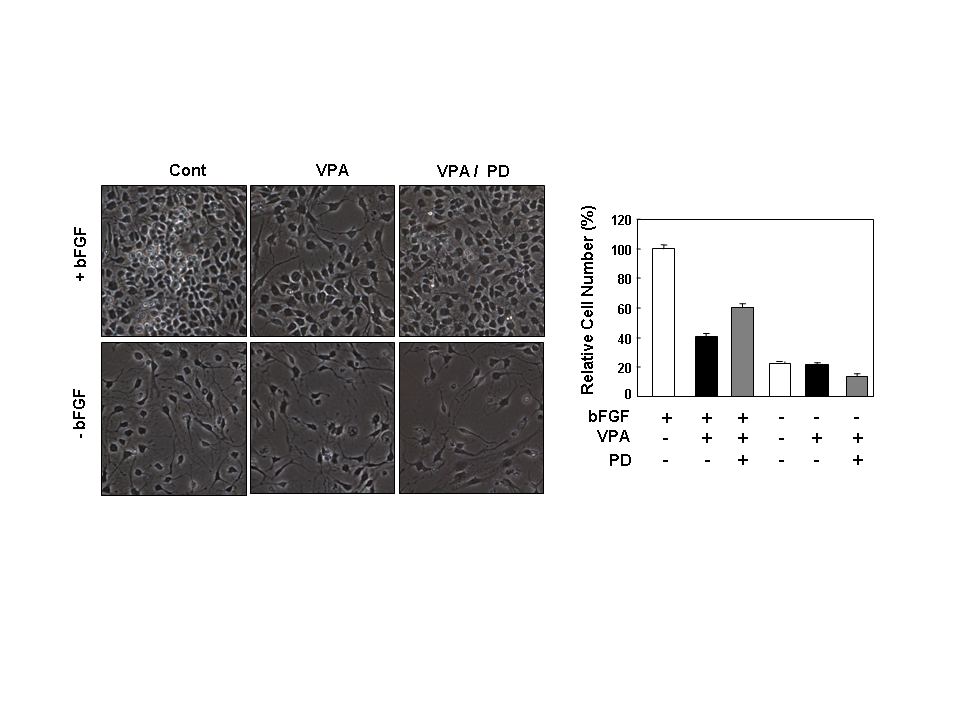

Supplement: Additional file 3 — Effects of MEK inhibitor PD98059 on the morphological differentiation and proliferation of NPCs. NPCs were grown in medium containing 10 ng/ml bFGF. Medium was then replaced with fresh medium containing 10 ng/ml bFGF, 20 μM PD98059, and/or 1 mM VPA, as indicated, and cultures were incubated for an additional 48 h. Cultures were photographed and relative numbers of cells were estimated as described in Figure 1. [file 1471-2121-9-66-S3.tiff]

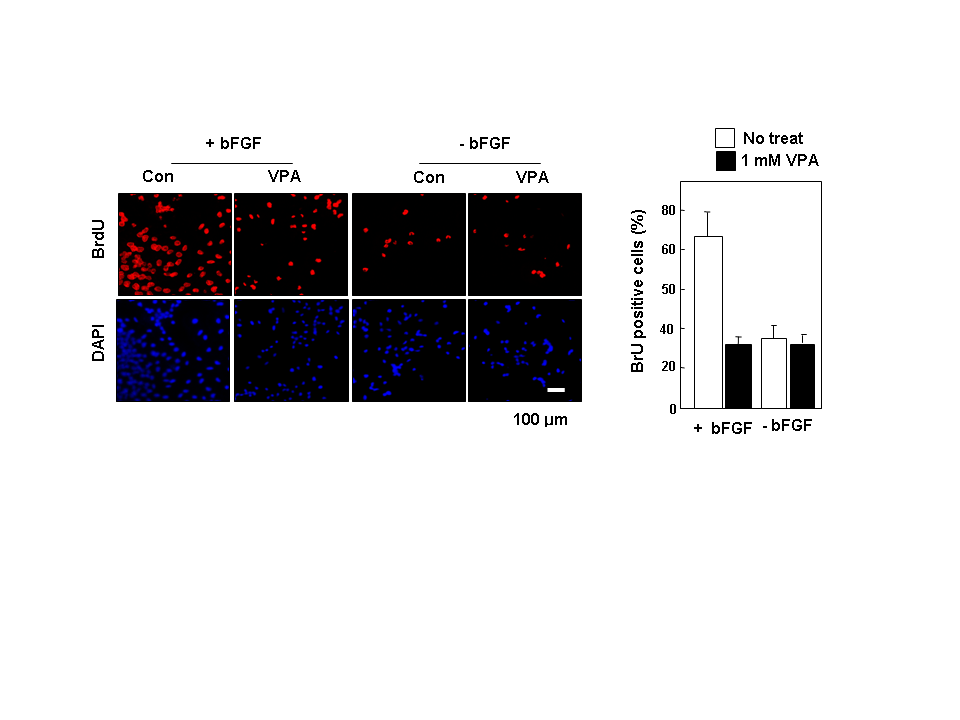

Supplement: Additional file 4 — Effects of bFGF and VPA on proliferation of NPCs. This experiment is similar to that shown in Figure 3B. Cells were processed for immunofluorescent labeling of BrdU (red). Nuclei were counterstained with DAPI (blue). Images are 100× magnifications. Right panel: Estimate of the percentage of BrdU-positive cells. Error bars indicate the standard deviation of three independent experiments. [file 1471-2121-9-66-S4.tiff]

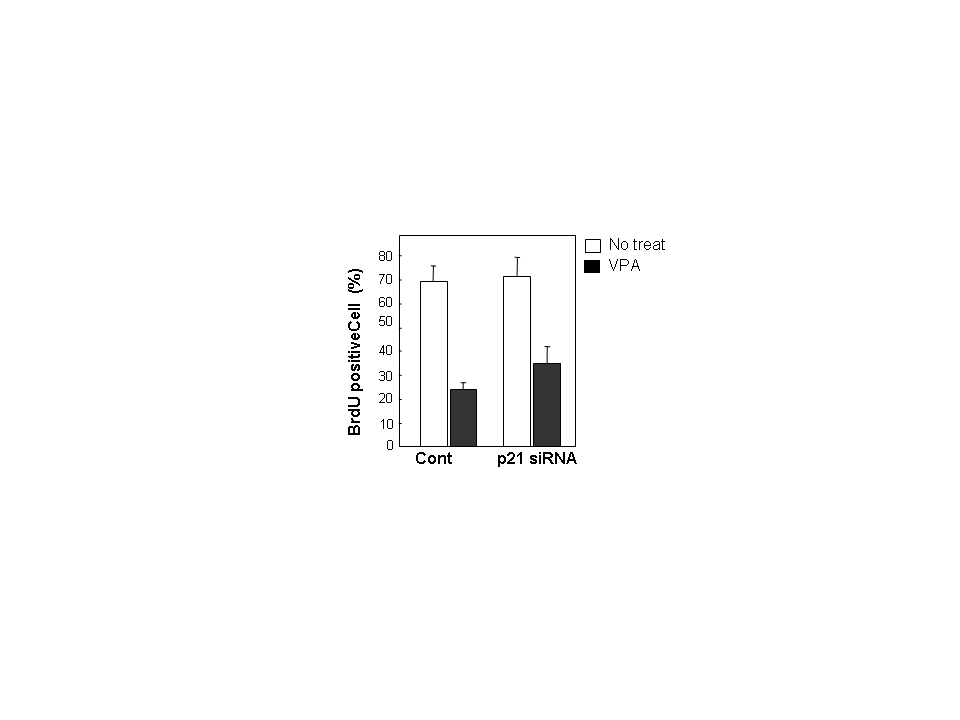

Supplement: Additional file 5 — Effects of p21Cip/WAF1 siRNA on VPA-induced inhibition of proliferation of NPCs grown in the presence of bFGF. This figure shows the quantitative data for Figure 3D (lower panel). Relative numbers of BrdU-positive cells were quantified. Error bars indicate the standard deviation of three independent experiments. [file 1471-2121-9-66-S5.tiff]

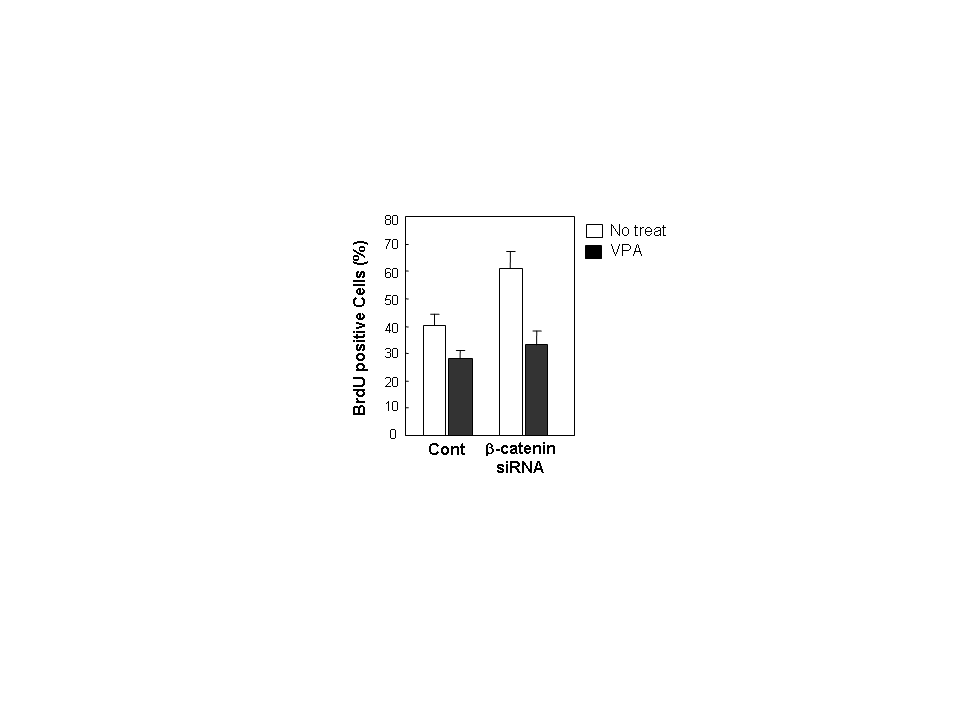

Supplement: Additional file 6 — Effects of ß-catenin siRNA on VPA-induced proliferation of NPCs grown in the presence of bFGF. This figure shows the quantitative data for Figure 5C. Relative numbers of BrdU-positive cells were quantified. Error bars indicate the standard deviation of three independent experiments. [file 1471-2121-9-66-S6.tiff]

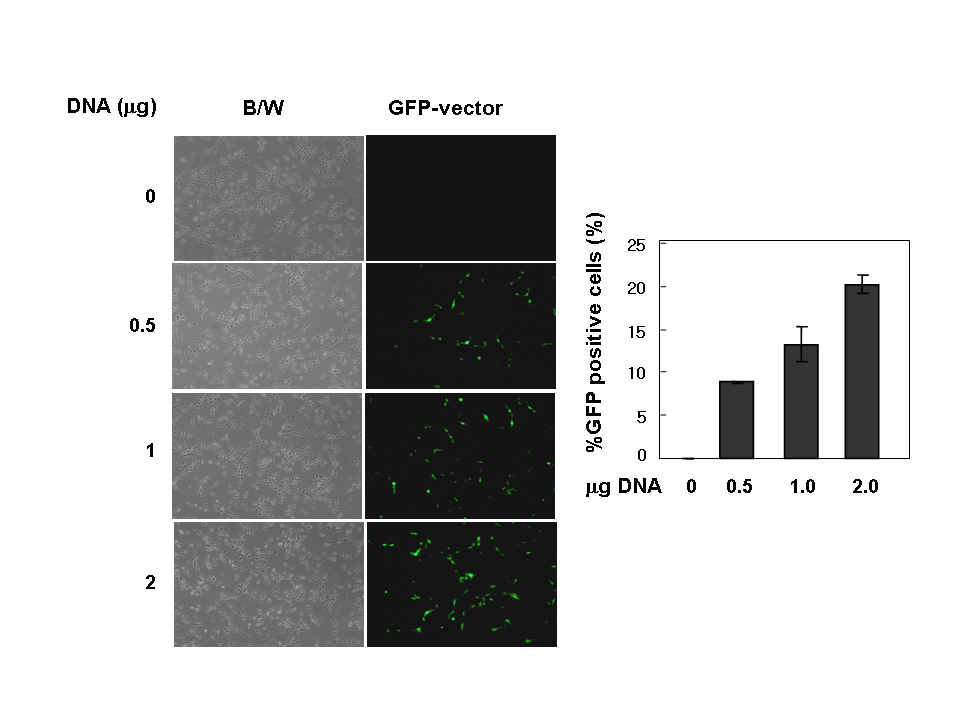

Supplement: Additional file 7 — Measurements of NPC transfection efficiencies. NPCs were grown in N2 medium containing bFGF (10 ng/ml), and transfected with 0, 0.5, 1, 2, or 3 µg pEGFP-C1 (Clontech) as described in Methods. Percentages of GFP-positive cells were estimated 24 h after transfection. Error bars indicate the standard deviation of three independent experiments. [file 1471-2121-9-66-S7.tiff]
